# Skeletal Muscle Density as an Independent Predictor of Prolonged Postoperative Hospital Stay After Surgery for Acute Cholecystitis

**DOI:** 10.3390/jcm15072473

**Published:** 2026-03-24

**Authors:** Hanbaro Kim, Min Ju Kim, Jeong Mok Lee, Han Zo Choi

**Affiliations:** 1 Department of Surgery, Kangnam Sacred Heart Hospital, Hallym University College of Medicine, Seoul 07441, Republic of Korea; alswn1216@hallym.or.kr (M.J.K.); jmok123@hallym.or.kr (J.M.L.); 2 Department of Emergency Medicine, Kyung Hee University Hospital at Gangdong, Kyung Hee University College of Medicine, Seoul 05278, Republic of Korea; sacehan@daum.net

**Keywords:** acute cholecystitis, skeletal muscle density, postoperative length of stay

## Abstract

**Background/Objectives**: Prolonged postoperative length of stay (LOS) is associated with increased morbidity and healthcare utilization following surgery for acute cholecystitis. The prognostic value of skeletal muscle density (SMD), a marker of muscle quality, is unclear. We aimed to evaluate the association between SMD and prolonged LOS and to compare the predictive performance of SMD with that of skeletal muscle index (SMI). **Methods**: A retrospective study of 382 patients who underwent surgery for acute cholecystitis was conducted. LOS was defined using mean- and median-based cut-offs. Multivariate logistic regression was used to identify independent predictors. Model performance was assessed using the area under the receiver operating characteristic curve (AUC) and Akaike information criterion (AIC). Robustness was assessed using count-based modeling, spline analysis, and model calibration. **Results**: Patients with prolonged LOS were older, had lower body mass index and serum albumin levels, higher inflammatory markers, and more comorbidities, and had significantly lower SMD. Higher SMD was independently associated with a reduced risk of prolonged LOS (adjusted OR per 1-HU increase, 0.93; 95% CI, 0.88–0.97; *p* = 0.002). The SMD-based model showed acceptable discrimination (AUC 0.78) and slightly better model fit than the SMI-based model (AIC 365.1 vs. 371.2). In secondary analyses, patients in the lowest SMD quartile had significantly higher postoperative complication rates than the remaining patients (10.5% vs. 2.8%; *p* = 0.004). **Conclusions**: Overall, lower SMD was independently associated with prolonged LOS after surgery for acute cholecystitis and may serve as a readily available imaging biomarker for perioperative risk stratification.

## 1. Introduction

Acute cholecystitis is a common surgical emergency, with laparoscopic cholecystectomy being the standard treatment [1]. Although most patients recover uneventfully, a substantial proportion experience prolonged postoperative length of stay (LOS), which is often driven by postoperative complications and delayed functional recovery, leading to greater healthcare utilization [2]. Early identification of patients at risk for delayed recovery is therefore clinically important, particularly in the emergency surgical setting [3].

Sarcopenia and frailty have emerged as key determinants of postoperative outcomes [4]. Most previous studies have focused on skeletal muscle index (SMI), a CT-based measure of muscle quantity; they have shown that reduced muscle mass is associated with worse surgical outcomes [5,6,7]. However, muscle quantity alone does not fully reflect muscle health. Skeletal muscle density (SMD), derived from CT attenuation values, reflects intramuscular fat infiltration and muscle quality, which are closely linked to metabolic dysfunction, inflammation, and physical performance [8]. In patients with cancer or critically ill patients, lower SMD is a stronger predictor of adverse outcomes than SMI [9,10]. However, despite growing evidence in oncologic and critically ill populations, the prognostic value of SMD in acute inflammatory surgical conditions, such as acute cholecystitis, remains largely unexplored.

Furthermore, most studies have evaluated outcomes using binary endpoints such as complications or mortality, rather than recovery-related outcomes such as LOS, which integrates postoperative complications, functional recovery, and healthcare utilization [11,12]. Whether muscle quality, as assessed by SMD, is independently associated with prolonged LOS and adds prognostic value over SMI remains unclear.

In this study, we aimed to evaluate the association between skeletal muscle density and prolonged postoperative LOS in patients undergoing surgery for acute cholecystitis and to compare the predictive performance of SMD with that of SMI using multivariate and sensitivity analyses.

## 2. Materials and Methods

### 2.1. Study Design and Population

**Figure 1:**
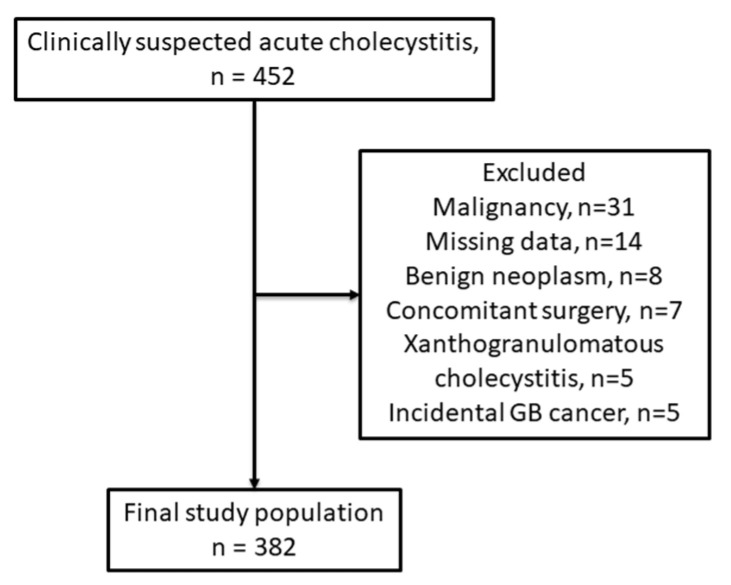
Flowchart of patient selection. A total of 452 patients who underwent surgery for clinically suspected acute cholecystitis were screened. After excluding patients with malignancy, concomitant surgery, benign or incidental gallbladder pathology, and missing data, 382 patients were included in the final analysis.

Between January 2016 and December 2023, a total of 452 consecutive patients who underwent surgery for clinically suspected acute cholecystitis were retrospectively screened. Patients with a history of malignancy (*n* = 31), those who underwent concomitant surgery in other departments (*n* = 7), as well as patients with benign gallbladder neoplasms (*n* = 8), xanthogranulomatous cholecystitis (*n* = 5), and incidental gallbladder cancer (*n* = 5) were excluded. Patients with missing key clinical or imaging data (*n* = 14) were also excluded (Figure 1). After these exclusions, 382 patients were included in the final analysis. Clinical, laboratory, operative, and postoperative outcome data were obtained from the institutional electronic medical record (EMR) system, and preoperative abdominal computed tomography images were retrieved for body composition analysis. This study was approved by the Institutional Review Board of Kangnam Sacred Heart Hospital, Hallym University College of Medicine (IRB No. 2025-11-028). The requirement for informed consent was waived because of the retrospective nature of the study.

### 2.2. Body Composition Assessment

**Figure 2:**
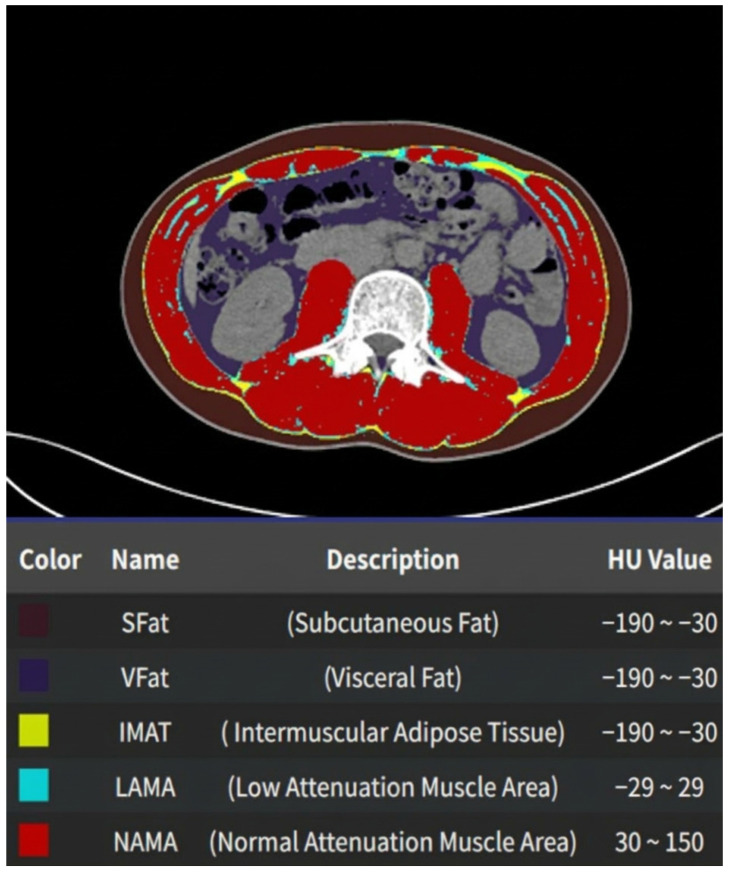
AI-based automated body composition analysis at the L3 vertebral level on computed tomography. Representative axial abdominal computed tomography (CT) image at the level of the third lumbar vertebra (L3) showing automated AI-based segmentation of body composition components. Skeletal muscle and adipose tissue compartments were identified and color-coded according to predefined Hounsfield unit (HU) thresholds. Subcutaneous fat (SFat), visceral fat (VFat), and intermuscular adipose tissue (IMAT) were defined within the range of −190 to −30 HU. Skeletal muscle was subdivided into low attenuation muscle area (LAMA; −29 to 29 HU), representing lipid-infiltrated muscle, and normal attenuation muscle area (NAMA; 30 to 150 HU), representing high-quality contractile muscle. Skeletal muscle area (SMA) was calculated as the sum of LAMA and NAMA, and skeletal muscle density (SMD) was derived as the mean HU value of the total segmented muscle area.

Body composition parameters were assessed using an artificial intelligence–based software AID-U™ (version 1.0.0, iIAD Inc., Seoul, Republic of Korea). On preoperative abdominal computed tomography images, subcutaneous fat area (SFA), visceral fat area (VFA), and skeletal muscle area (SMA) were automatically quantified at the level of the third lumbar vertebra (L3) (Figure 2). SMA was defined as the sum of low-attenuation muscle area (LAMA) and normal-attenuation muscle area (NAMA) within the segmented skeletal muscle compartment. SMD was calculated as the mean Hounsfield unit (HU) value of the entire segmented SMA, reflecting intramuscular fat infiltration and muscle quality. SMI was calculated by normalizing SMA to height squared (cm^2^/m^2^).

### 2.3. Definition of Postoperative Length of Stay and Outcome Variables

Patients were discharged when they met all of the following criteria: (1) adequate pain control with oral analgesics, (2) tolerance of regular diet without nausea or vomiting, (3) independent ambulation, (4) absence of fever or signs of infection, (5) stable vital signs, and (6) absence of complications requiring further in-hospital management. The discharge decision was made by the attending surgeon in consultation with the surgical team.

Postoperative length of stay (LOS) was defined as the number of days from the date of surgery to the date of hospital discharge and was used as an integrated indicator of postoperative recovery, encompassing functional recovery, complications, and healthcare utilization.

For the primary analysis, LOS was dichotomized using the mean LOS (4.52 days) as the cut-off, with patients exceeding this threshold classified as having prolonged LOS. To ensure robustness and account for potential effects of institutional or non-clinical factors on discharge timing, sensitivity analyses were performed using (1) the median LOS (3 days) as an alternative cut-off and (2) LOS modeled as a continuous variable using negative binomial regression.

### 2.4. Secondary Outcome Analysis

Postoperative complications were analyzed as a secondary outcome. Given the limited number of complication events, analyses were restricted to univariate logistic regression and quartile-based comparisons using chi-square or Fisher’s exact tests, as appropriate, to avoid model overfitting.

### 2.5. Statistical Analysis

Continuous variables were summarized as medians with interquartile ranges or means with standard deviations, depending on distributional characteristics. Categorical variables were presented as frequencies and percentages. Group comparisons were performed using the Mann–Whitney U test for continuous variables and the chi-square test or Fisher’s exact test for categorical variables, as appropriate.

Multivariate logistic regression was used to identify independent predictors of prolonged LOS. Covariates were selected a priori based on clinical relevance and prior literature and included age, sex, body mass index, serum albumin, blood urea nitrogen, hypertension, diabetes mellitus, and chronic kidney disease. Postoperative complications and surgical approach were excluded because they occur after the exposure (SMD) and lie on the causal pathway between muscle quality and postoperative recovery. Multicollinearity was assessed using variance inflation factors, and all included variables demonstrated acceptable levels.

To evaluate model robustness, heteroskedasticity-consistent robust standard errors (HC3) were applied. As an additional sensitivity analysis addressing potential information loss owing to dichotomization, negative binomial regression was performed with LOS retained as a continuous outcome, and effect estimates were expressed as incidence rate ratios with 95% confidence intervals.

The linearity assumption between SMD and the log-odds of prolonged LOS was assessed using restricted cubic spline functions with four knots. Overall and nonlinear components were evaluated using Wald tests. Prespecified subgroup analyses were conducted according to age (<65 vs. ≥65 years) and the absence of postoperative complications. Interaction terms were examined, and the analyses were considered exploratory.

Model discrimination was evaluated using the AUC, and model fit was compared using Akaike’s Information Criterion (AIC). Calibration was assessed using bootstrap-based calibration curves and the Hosmer–Lemeshow test.

All statistical analyses were conducted using R software (version 4.5.0; R Foundation for Statistical Computing, Vienna, Austria). A two-sided *p*-value < 0.05 was considered statistically significant.

## 3. Results

### 3.1. Baseline Characteristics According to Postoperative Length of Stay

A total of 382 patients were included in the analysis. The mean LOS was 4.52 days, and the median LOS was 3 days. Patients were categorized into prolonged and non-prolonged LOS groups using both mean- and median-based cut-offs.

When stratified by the mean LOS cut-off, patients with prolonged LOS were significantly older (median 71.5 vs. 58.0 years, *p* < 0.001) and had a lower body mass index (median 24.0 vs. 25.6 kg/m^2^, *p* < 0.001) compared with those without prolonged LOS. The prolonged LOS group also showed lower serum albumin levels (median 3.8 vs. 4.2 g/dL, *p* < 0.001) and higher blood urea nitrogen (median 18.0 vs. 14.4 mg/dL, *p* < 0.001) and C-reactive protein levels (median 84.0 vs. 24.8 mg/L, *p* = 0.010). Diabetes mellitus (*p* = 0.006) and chronic kidney disease (*p* = 0.003) were more prevalent in the prolonged LOS group. Percutaneous transhepatic gallbladder drainage (*p* < 0.001) and open surgery (*p* < 0.001) were more frequently performed in patients with prolonged LOS, and postoperative complications were observed exclusively in this group (*p* < 0.001). With respect to body composition, SMD (median 27.6 vs. 35.4 HU, *p* < 0.001) and SMI (median 39.7 vs. 46.6 cm^2^/m^2^, *p* < 0.001) were significantly lower in the prolonged LOS group, whereas visceral fat area did not differ between groups (*p* = 0.615).

Similar patterns were observed when patients were stratified by the median LOS cut-off, with consistent differences in age, laboratory parameters, comorbidities, procedural characteristics, and SMD. Detailed baseline characteristics according to both cut-offs are presented in Table 1 and Supplementary Table S1.

### 3.2. Multivariate Logistic Regression Analysis for Prolonged Postoperative Length of Stay

In multivariate logistic regression analysis using the mean-based LOS cut-off, higher SMD was independently associated with a lower risk of prolonged LOS after adjustment for age, sex, body mass index, serum albumin, blood urea nitrogen, hypertension, diabetes mellitus, and chronic kidney disease (adjusted odds ratio [OR] per 1-HU increase, 0.93; 95% confidence interval [CI], 0.88–0.97; *p* = 0.002). When expressed per standard deviation increase (8.5 HU), SMD was associated with a 36% reduction in the risk of prolonged LOS (OR, 0.64; 95% CI, 0.46–0.90). Lower body mass index (OR, 0.90; 95% CI, 0.83–0.97; *p* = 0.010) and lower serum albumin levels (OR, 0.51; 95% CI, 0.27–0.97; *p* = 0.039) were also independently associated with prolonged LOS, whereas chronic kidney disease was associated with an increased risk (OR, 3.12; 95% CI, 1.05–9.43; *p* = 0.040).

When the outcome was defined using the median LOS cut-off, SMD remained significantly associated with prolonged LOS (OR, 0.96; 95% CI, 0.92–1.00; *p* = 0.038), demonstrating consistency across alternative definitions of prolonged hospitalization. Robust standard error estimation yielded similar effect estimates for SMD (robust OR, 0.93; 95% CI, 0.88–0.97), supporting the stability of the primary findings. Detailed results of the multivariate analyses are shown in Table 2.

### 3.3. Model Performance and Comparison with Alternative Muscle Indices

**Figure 3:**
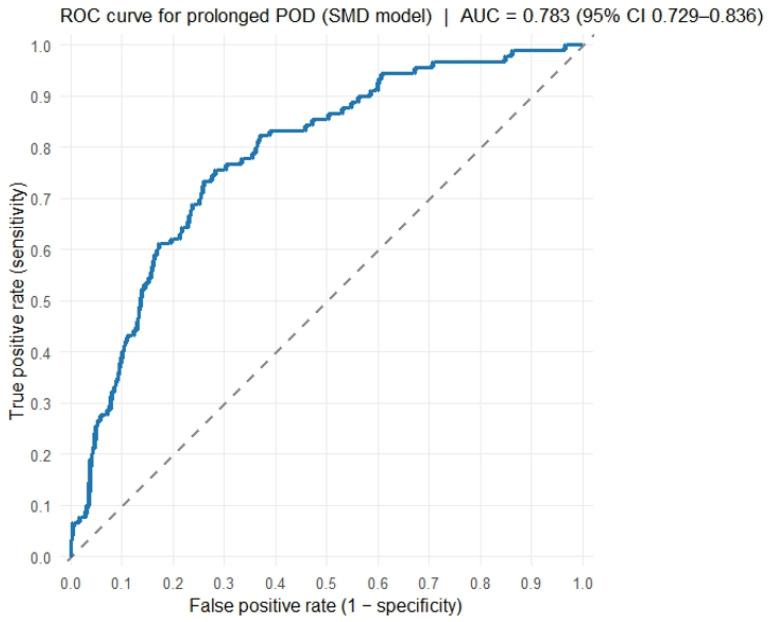
Receiver operating characteristic curve (SMD-based model). Receiver operating characteristic (ROC) curve of the skeletal muscle density–based multivariate model for predicting prolonged postoperative length of stay. The area under the curve was 0.78, indicating acceptable discriminative performance.

When skeletal muscle index was substituted for skeletal muscle density in the multivariable model, SMI was not independently associated with prolonged LOS after covariate adjustment (adjusted OR per 1-unit increase, 0.96; 95% CI, 0.92–1.00; *p* = 0.066) (Supplementary Table S2). By contrast, the discriminative performance of the SMD–based model was acceptable, with an area under the receiver operating characteristic curve (AUC) of 0.78. Compared with a model that uses SMI instead of SMD, the SMD–based model demonstrated superior performance, as reflected by lower Akaike information criterion (AIC; 365.1 vs. 371.2) and higher AUC (0.78 vs. 0.77). These findings support the use of SMD as a clinically informative body composition metric in subsequent analyses. Model performance metrics are summarized in Table 3, and the receiver operating characteristic curve is shown in Figure 3.

### 3.4. Sensitivity Analyses

In a sensitivity analysis treating LOS as a continuous count outcome using negative binomial regression, higher SMD remained significantly associated with shorter postoperative LOS after multivariate adjustment (incidence rate ratio [IRR], 0.97; 95% CI, 0.96–0.98; *p* < 0.001). This finding indicates that the observed association was not driven by LOS dichotomization and was robust across different modeling approaches. Results of the negative binomial regression analysis are presented in Supplementary Table S3.

### 3.5. Secondary Outcome: Postoperative Complications

Postoperative complications occurred in 18 patients (4.7%). Complication rates increased progressively across SMD quartiles, with the highest rate observed in the lowest quartile (10.5%) compared with the remaining patients (2.8%; Fisher’s exact *p* = 0.0045).

In univariate logistic regression analysis, lower SMD was significantly associated with an increased risk of postoperative complications (OR per 1-HU increase, 0.91; 95% CI, 0.87–0.96; *p* < 0.001). Given the limited number of events, multivariable adjustment was restricted to a minimal model, and the association was attenuated after adjustment for age and serum albumin.

### 3.6. Assessment of Linearity and Subgroup Analyses

Restricted cubic spline analysis demonstrated a significant overall association between SMD and prolonged LOS (Wald test for overall effect, *p* = 0.021). By contrast, the nonlinear component was not significant (*p* = 0.752), indicating an approximately linear inverse relationship across the observed range of SMD. Detailed spline coefficients are provided in Supplementary Table S4, and the spline curve is shown in Supplementary Figure S1.

Subgroup analyses showed that the inverse association between SMD and prolonged LOS was directionally consistent across predefined subgroups, including patients aged ≥65 years (OR, 0.93; 95% CI, 0.87–0.99) and those without postoperative complications (OR, 0.94; 95% CI, 0.89–0.99). No significant interactions were observed. Results of subgroup analyses are summarized in Table 4.

Odds ratios were derived from multivariable logistic regression models adjusted for age, sex, body mass index, albumin, blood urea nitrogen, hypertension, diabetes mellitus, and chronic kidney disease.

### 3.7. Model Calibration

Calibration assessment using bootstrap resampling demonstrated good agreement between predicted and observed probabilities of prolonged LOS, with a low mean absolute error of 0.021, indicating satisfactory model calibration. The calibration plot is presented in Supplementary Figure S2.

## 4. Discussion

This study demonstrates that preoperative skeletal muscle density (SMD), a marker of muscle quality, was independently associated with postoperative recovery in patients undergoing cholecystectomy for acute cholecystitis. Lower SMD was consistently associated with prolonged LOS across multiple analytic approaches, including multivariable logistic regression using different LOS cut-offs, negative binomial regression treating LOS as a continuous outcome, and sensitivity analyses with robust standard errors. In addition, models incorporating SMD showed modestly improved predictive performance compared with SMI, as indicated by AUC and AIC, suggesting that muscle quality may provide greater prognostic value than muscle quantity alone in this clinical setting.

Importantly, the inverse association between SMD and prolonged LOS persisted even among patients without postoperative complications. This finding suggests that skeletal muscle density may serve as a marker of intrinsic physiological reserve, reflecting the patient’s capacity to tolerate surgical stress and recover after surgery beyond the occurrence of overt complications. The magnitude of association was clinically meaningful, corresponding to approximately a 36% reduction in the risk of a prolonged postoperative stay per standard deviation increase in SMD.

Beyond its impact on LOS independent of complications, lower SMD was also associated with a higher incidence of postoperative complications, particularly among patients in the lowest SMD quartile. Although the number of complication events was limited, this observation further supports the role of impaired muscle quality as a marker of perioperative vulnerability.

Previous studies have extensively documented the prognostic significance of body composition, particularly sarcopenia, in oncologic surgery [13,14,15]. In patients with gastrointestinal and hepatobiliary malignancies, reduced SMI and low SMD have been associated with increased postoperative complications, longer hospital stays, and inferior survival [16,17]. Beyond malignancy, emerging evidence suggests that impaired muscle reserve adversely affects outcomes in non-oncologic conditions such as chronic liver disease, cardiovascular disease, trauma, and critical illness [18,19,20,21]. However, data regarding the impact of body composition on postoperative recovery in benign acute surgical diseases have been limited. Our findings extend the existing literature by demonstrating that muscle quality, as assessed by SMD, is an important determinant of postoperative recovery even in a common benign condition traditionally considered low-risk.

Acute cholecystitis represents a unique clinical scenario characterized by an acute inflammatory insult superimposed on the patient’s baseline physiological reserve. In this context, skeletal muscle plays a critical role as a metabolic, immunologic, and functional reserve during systemic stress [22,23]. Reduced SMD reflects increased intramuscular fat infiltration and is associated with impaired insulin sensitivity, chronic low-grade inflammation, and diminished mitochondrial function [24,25]. These alterations may compromise the patient’s ability to tolerate surgical stress and resolve postoperative inflammation, thereby contributing to delayed recovery and prolonged hospitalization. The observed linear inverse relationship between SMD and prolonged LOS in our spline analysis supports a dose–response association rather than a threshold effect.

Notably, SMD remained independently associated with postoperative recovery after adjustment for age, body mass index, nutritional markers, renal function, and major comorbidities. By contrast, SMI demonstrated inferior predictive performance in model comparison analyses. These findings suggest that muscle quality captures clinically relevant vulnerability not adequately reflected by muscle mass alone. In patients with acute cholecystitis, who often undergo preoperative CT as part of routine diagnostic workup, assessment of SMD may therefore provide an objective and readily available risk-stratification tool.

## 5. Conclusions

The clinical implications of our findings are potentially significant. Identification of patients at a higher risk for prolonged postoperative recovery based on preoperative SMD may facilitate individualized perioperative management, including closer postoperative monitoring, early mobilization strategies, nutritional optimization, and discharge planning. From a health system perspective, incorporation of muscle quality metrics into perioperative risk assessment models could improve resource allocation and bed management, particularly in high-volume emergency surgical settings. Although early cholecystectomy for acute cholecystitis is frequently performed in urgent settings where prehabilitation is not feasible, preoperative SMD assessment may still offer clinically meaningful information. Rather than serving as a tool for preoperative intervention, SMD may assist in perioperative risk stratification by identifying vulnerable patients who may benefit from intensified postoperative monitoring, early nutritional support, and individualized discharge planning.

This study has some limitations. First, the retrospective design and single-center setting may limit generalizability. The severity of acute cholecystitis was not formally classified according to the Tokyo Guidelines 2018 (TG18) [26]. Although proxies such as PTGBD placement, perforation, and gangrenous cholecystitis partially reflected disease severity, standardized TG18 grading was not available due to the retrospective nature of the dataset. Future studies should evaluate whether skeletal muscle density provides additional prognostic value within specific TG18 severity categories. Second, although LOS was used as a clinically relevant surrogate for postoperative recovery, it may be influenced by institutional practices and non-clinical factors. The mean LOS of 4.5 days in our cohort should be interpreted within the Korean healthcare context, where longer postoperative observation periods are standard due to comprehensive National Health Insurance coverage, cultural preferences for conservative postoperative care, and institutional protocols prioritizing patient safety. This underscores the importance of including objective outcome measures, such as complications, in future studies. Third, body composition was assessed at a single time point, precluding evaluation of dynamic changes in muscle quality. Finally, although we adjusted for multiple confounders, residual confounding cannot be completely excluded.

Despite these limitations, this study has notable strengths, including a relatively large cohort, comprehensive body composition analysis using routinely obtained CT images, and robust statistical validation across multiple modeling strategies. In conclusion, SMD appears to be an independent and clinically meaningful predictor of postoperative recovery after cholecystectomy for acute cholecystitis and outperforms conventional muscle mass–based indices. These findings highlight the importance of muscle quality in benign acute surgical diseases and support the integration of body composition assessment into perioperative risk stratification.

## Figures and Tables

**Table 1:** Baseline characteristics according to LOS group (mean-based cut-off).

Variable	Low LOS (≤Mean, N = 292)	High LOS (>Mean, N = 90)	*p*-Value
**Demographics and Vital Signs**			
Age, years	58.0 (45.0–68.5)	71.5 (60.0–79.0)	<0.001
Sex—Male	112 (38.4)	39 (43.3)	0.471
BMI, kg/m^2^	25.6 (23.4–28.2)	24.0(22.0–26.5)	<0.001
Initial SBP, mmHg	120.0 (110.0–140.0)	122.0 (110.0–140.0)	0.558
Initial DBP, mmHg	80.0 (70.0–80.0)	80.0 (70.0–80.0)	0.682
Initial MAP, mmHg	93.9 (86.7–99.2)	93.3 (86.7–100.0)	0.541
Initial HR, beats/min	80 (70–90)	80 (74–90)	0.123
Initial BT, °C	36.6 (36.4–36.7)	36.6 (36.3–36.9)	0.156
O_2_ saturation	99.0 (99.0–99.0)	99.0 (99.0–99.0)	0.680
**Laboratory Tests**			
WBC, ×10^3^/µL	10.6 (7.9–14.3)	10.8 (8.4–15.0)	0.585
Platelet, ×10^3^/µL	218.5 (184.0–272.0)	239.5 (190.0–289.0)	0.066
Albumin, g/dL	4.2 (3.9–4.4)	3.8 (3.4–4.1)	<0.001
BUN, mg/dL	14.4 (10.9–18.5)	18.0 (12.2–26.4)	<0.001
Creatinine, mg/dL	0.8 (0.7–1.0)	0.8 (0.7–1.1)	0.374
Total bilirubin, mg/dL	1.0 (0.7–1.7)	1.2 (0.7–2.1)	0.105
CRP, mg/L	24.8 (2.4–122.3)	84.0 (5.5–179.1)	0.010
**Comorbidities**			
Hypertension	109 (37.5)	46 (51.1)	0.029
Diabetes mellitus	66 (22.6)	34 (37.8)	0.006
Heart failure and arrhythmia	24 (8.2)	14 (15.6)	0.067
Liver cirrhosis	26 (8.9)	12 (13.3)	0.305
COPD	1 (0.3)	2 (2.2)	0.279
CKD	8 (2.7)	10 (11.1)	0.003
CVA	1 (0.3)	2 (2.2)	0.279
**Disease Severity**			
Gallstone	217 (74.3)	63 (70.0)	0.501
Perforation	21 (7.2)	12 (13.3)	0.110
PTGBD	83 (28.4)	50 (55.6)	<0.001
Open conversion	0 (0.0)	7 (7.8)	<0.001
**Type of cholecystitis**			0.290
Acute cholecystitis	188 (64.4)	54 (60.0)	
Gangrenous cholecystitis	95 (32.5)	30 (33.3)	
Acute cholecystitis with abscess	9 (3.1)	6 (6.7)	
**Body composition**			
SFA	148.3 (106.5–209.9)	125.1 (94.8–174.7)	0.005
VFA	173.6 (124.2–228.7)	169.7 (123.0–216.4)	0.615
SMD	35.4 (29.9–40.2)	27.6 (22.2–34.0)	<0.001
SMI	46.6 (40.1–53.4)	39.7 (33.8–47.6)	<0.001
Sarcopenia	66 (22.6)	39 (43.3)	<0.001
**Outcomes**			
Complication	0 (0)	18(20.0%)	<0.001
ICU Admission	25 (8.6%)	6 (6.7%)	0.723

LOS, postoperative length of stay; BMI, body mass index; SBP, systolic blood pressure; DBP, diastolic blood pressure; MAP, mean arterial pressure; HR, heart rate; BT, body temperature; WBC, white blood cell count; BUN, blood urea nitrogen; CRP, C-reactive protein; COPD, chronic obstructive pulmonary disease; CKD, chronic kidney disease; CVA, cerebrovascular accident; PTGBD, percutaneous transhepatic gallbladder drainage; SFA, subcutaneous fat area; VFA, visceral fat area; SMD, skeletal muscle density; SMI, skeletal muscle index; ICU, intensive care unit. Baseline demographic, clinical, laboratory, and body composition characteristics of patients according to LOS, dichotomized using the mean LOS value (4.52 days). Continuous variables are presented as median [interquartile range], and categorical variables as number (percentage).

**Table 2:** Multivariate logistic regression analyses for prolonged LOS (mean- and median-based cut-offs).

Variable	Mean-Based OR (95% CI)	*p*-Value	Median-Based OR (95% CI)	*p*-Value	Robust OR (95% CI)
SMD	0.93 (0.88–0.97)	0.002	0.96 (0.92–1.00)	0.038	0.93 (0.88–0.97)
Age	1.00 (0.98–1.03)	0.988	1.01 (0.99–1.03)	0.249	1.00 (0.97–1.03)
Sex (male)	0.69 (0.36–1.31)	0.251	0.87 (0.51–1.46)	0.590	0.69 (0.36–1.31)
BMI	0.90 (0.83–0.97)	0.010	0.97 (0.91–1.03)	0.370	0.90 (0.83–0.98)
Albumin	0.51 (0.27–0.97)	0.039	1.02 (0.99–1.05)	0.409	0.51 (0.27–0.97)
BUN	1.01 (0.99–1.03)	0.223	1.00 (0.98–1.02)	0.726	1.01 (0.99–1.03)
Hypertension	1.06 (0.61–1.83)	0.833	1.09 (0.69–1.72)	0.711	1.06 (0.60–1.88)
Diabetes mellitus	1.56 (0.88–2.77)	0.127	1.48 (0.90–2.45)	0.124	1.56 (0.85–2.87)
CKD	3.12 (1.05–9.43)	0.040	3.00 (1.04–9.93)	0.051	3.12 (0.77–12.68)

SMD, skeletal muscle density; BMI, body mass index; BUN, blood urea nitrogen; CKD, chronic kidney disease. Prolonged LOS was defined using both mean-based and median-based cut-offs to assess the robustness of the findings. Robust standard errors (HC3) were applied as a sensitivity analysis for the mean-based model; therefore, only ORs and 95% CIs are reported for the robust model without corresponding *p*-values. An OR < 1 indicates a decreased risk of prolonged LOS, whereas an OR > 1 indicates an increased risk.

**Table 3:** Comparison of model performance between SMD- and SMI-based multivariable models for prolonged LOS.

Model	Variables Included	AIC	AUC (ROC)
SMD model	SMD, age, sex, BMI, albumin, BUN, hypertension, diabetes mellitus, chronic kidney disease	365.1	0.783
SMI model	SMI, age, sex, BMI, albumin, BUN, hypertension, diabetes mellitus, chronic kidney disease	371.2	0.769

SMD, skeletal muscle density; SMI, skeletal muscle index; AIC, Akaike information criterion; AUC, area under the curve; ROC, receiver operating characteristic; BMI, body mass index; BUN, blood urea nitrogen.

**Table 4:** Subgroup analyses of the association between skeletal muscle density and prolonged postoperative length of stay.

Subgroup	Odds Ratio	95% CI	*p*-Value
Overall	0.93	0.88–0.97	0.002
Age < 65 years	0.95	0.87–1.04	0.294
Age ≥ 65 years	0.93	0.87–0.99	0.027
No complication	0.94	0.89–0.99	0.017

## Data Availability

The data presented in this study are available on request from the corresponding author.

## Supplementary Materials

The following supporting information can be downloaded at https://www.mdpi.com/article/10.3390/jcm15072473/s1. Figure S1: Restricted cubic spline curve for skeletal muscle density; Figure S2: Calibration plot; Table S1: Baseline characteristics according to LOS group (median-based cut-off); Table S2: Sensitivity analysis using skeletal muscle index (SMI) instead of skeletal muscle density in the multivariable logistic regression model, Table S3: Negative binomial regression analysis for postoperative length of stay (LOS); Table S4: Restricted cubic spline analysis of skeletal muscle density for prolonged postoperative length of stay.

## Abbreviations

The following abbreviations are used in this manuscript:

LOS	Length of stay
SMD	Skeletal muscle density
SMI	Skeletal muscle index
