# Supplementary material for: Skeletal Muscle Density as an Independent Predictor of Prolonged Postoperative Hospital Stay After Surgery for Acute Cholecystitis
Source: J Clin Med. 2026 Mar 24;15(7):2473. doi: 10.3390/jcm15072473 (PMC13072955; doi:10.3390/jcm15072473)
Supplement: Supplementary file 1 [file jcm-15-02473-s001.zip › jcm-4210087-supplementary.pdf]

Supplementary Figure S1. Restricted cubic spline curve for skeletal muscle density

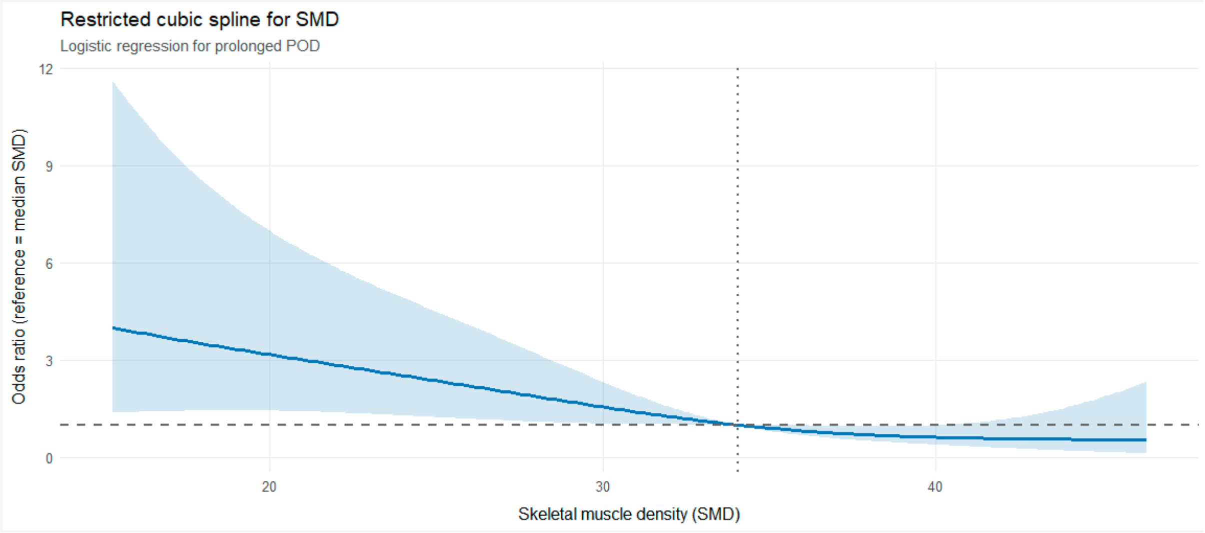

Restricted cubic spline curve depicting the association between skeletal muscle density and the predicted probability of prolonged postoperative length of stay. Shaded areas represent 95% confidence intervals.

Supplementary Figure S2. Calibration plot

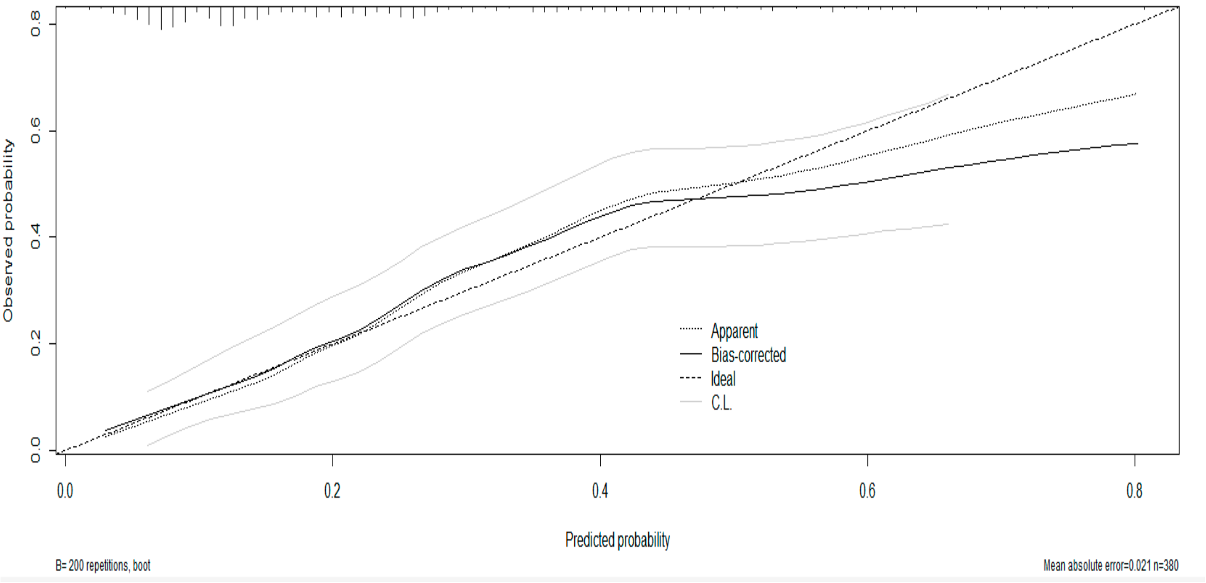

Calibration plot of the skeletal muscle density–based multivariable logistic regression model generated using bootstrap resampling (200 repetitions). The bias-corrected curve demonstrates agreement between predicted and observed probabilities.

Supplementary Table S1. Baseline characteristics according to LOS group (median-based cut-off)

Variable	Low (≤ median, N=227)	High LOS (> median, N=155)	*p*-Value
**Demographics and Vital Signs**			
Age, years	58.0 (44.0–67.0)	68.0 (53.5–77.0)	<0.001
Sex (male)	87 (38.3)	64 (41.3)	0.635
BMI	25.4 (23.4–28.0)	24.8 (22.7–27.3)	0.064
Initial SBP, mmHg	120.0 (110.0–131.5)	130.0 (110.0–140.0)	0.259
Initial DBP, mmHg	80.0 (70.0–80.0)	80.0 (70.0–80.0)	0.744
Initial MAP, mmHg	93.3 (86.7–97.3)	93.3 (86.7–100.0)	0.213
Initial HR, beats/min	80.0 (70.0–90.0)	80.0 (76.5–90.0)	0.024
Initial BT, °C	36.6 (36.4–36.7)	36.6 (36.4–36.8)	0.013
O2 saturation	99.0 (99.0–99.0)	99.0 (99.0–99.0)	0.111
**Laboratory Tests**			
WBC, $\times 10^3/\mu L$	10.5 (7.9–14.1)	11.5 (8.6–15.5)	0.083
Platelet, $\times 10^3/\mu L$	214.0 (174.0–263.0)	240.0 (193.5–295.0)	0.001
Albumin, g/dL	4.1 (3.9–4.4)	4.0 (3.5–4.3)	<0.001
BUN, mg/dL	14.2 (10.9–18.5)	16.0 (12.1–22.1)	0.004
Creatinine, mg/dL	0.8 (0.7–1.0)	0.8 (0.7–1.0)	0.237
Total bilirubin, mg/dL	1.0 (0.7–1.6)	1.1 (0.7–1.9)	0.280
CRP, mg/L	23.1 (2.1–107.2)	57.7 (5.8–177.2)	0.002
**Comorbidities**			
Hypertension	83 (36.6)	72 (46.8)	0.060
Diabetes Mellitus	14 (6.2)	24 (15.5)	0.005
Heart failure & arrhythmia	48 (21.1)	52 (33.5)	0.010
Liver cirrhosis	23 (10.1)	15 (9.7)	1.000
COPD	1 (0.4)	2 (1.3)	0.739
CKD	5 (2.2)	13 (8.4)	0.011
CVA	1 (0.4)	2 (1.3)	0.739
**Disease Severity**			
Gallstone	171 (75.3)	109 (70.3)	0.333
Perforation	13 (5.7)	20 (12.9)	0.023
PTGBD	53 (23.3)	80 (51.6)	<0.001

Open conversion	0 (0.0)	7 (4.5)	0.004
**Type of cholecystitis**			0.138
Acute cholecystitis	153 (67.4)	89 (57.4)	
Gangrenous cholecystitis	66 (29.1)	59 (38.1)	
Acute cholecystitis with abscess	8 (3.5)	7 (4.5)	
**Body composition**			
SFA	147.8 (105.4–207.7)	131.8 (101.9–184.2)	0.130
VFA	173.5 (123.8–224.3)	171.7 (124.8–231.0)	0.886
SMD	35.4 (29.9–40.4)	30.7 (25.0–38.1)	<0.001
SMI	46.5 (40.2–52.6)	43.5 (36.7–50.5)	0.007
Sarcopenia	51 (22.5)	54 (34.8)	0.011
**Others**			
Complication	0 (0.0)	18 (11.6)	<0.001
ICU Admission	17 (7.5)	14 (9.0)	0.725

LOS, postoperative length of stay; BMI, body mass index; SBP, systolic blood pressure; DBP, diastolic blood pressure; MAP, mean arterial pressure; HR, heart rate; BT, body temperature; WBC, white blood cell count; BUN, blood urea nitrogen; CRP, C-reactive protein; COPD, chronic obstructive pulmonary disease; CKD, chronic kidney disease; CVA, cerebrovascular accident; PTGBD, percutaneous transhepatic gallbladder drainage; SFA, subcutaneous fat area; VFA, visceral fat area; SMD, skeletal muscle density; SMI, skeletal muscle index; ICU, intensive care unit.

Baseline demographic, clinical, laboratory, and body composition characteristics of patients according to LOS, dichotomized using the median LOS value. Continuous variables are presented as median [interquartile range], and categorical variables as number (percentage).

Supplementary Table S2. Sensitivity analysis using SMI instead of skeletal muscle density in the multivariable logistic regression model

Variable	Adjusted OR	95% CI	*p*-Value
SMI (per unit)	0.96	0.92–1.00	0.066
Age (per year)	1.02	1.00–1.04	0.129
Sex (male)	0.80	0.41–1.55	0.517
BMI (kg/m²)	0.97	0.88–1.06	0.458
Albumin (g/dL)	0.43	0.23–0.79	0.007
BUN (mg/dL)	1.01	0.99–1.03	0.299

Hypertension	1.11	0.65–1.91	0.700
Diabetes Mellitus	1.72	0.97–3.06	0.063
CKD	3.87	1.34–11.57	0.013

SMI, skeletal muscle index; ORs, odds ratios; CIs, confidence intervals; BMI, body mass index; BUN, blood urea nitrogen; CKD, chronic kidney disease.

SMI is expressed per 1-unit increase. Age is expressed per 1-year increase. BMI is expressed per 1 kg/m² increase. Albumin and BUN are expressed per 1-unit (g/dL and mg/dL, respectively) increase. Sex (male), hypertension, diabetes mellitus, and CKD are binary variables.

Supplementary Table S3. Negative binomial regression analysis for LOS

Variable	IRR	95% CI	*p*-Value
SMD (per unit)	0.97	0.96–0.98	<0.001
Age (per year)	1.00	1.00–1.01	0.338
Sex (male)	0.86	0.72–1.01	0.068
BMI (kg/m²)	0.98	0.96–1.00	0.026
Albumin (g/dL)	1.00	0.99–1.01	0.827
BUN (mg/dL)	1.01	1.01–1.02	<0.001
Hypertension	1.12	0.97–1.31	0.118
Diabetes mellitus	1.00	0.85–1.18	0.989
Chronic kidney disease	0.98	0.72–1.36	0.925

LOS, postoperative length of stay; IRRs, incidence rate ratios; CIs, confidence intervals; SMD, skeletal muscle density; BMI, body mass index; BUN, blood urea nitrogen

SDM, BMI, albumin, and BUN are expressed per 1-unit increase; age is expressed per 1-year increase; sex (male), hypertension, diabetes mellitus, and chronic kidney disease are binary variables. Results are presented as IRRs with 95% CIs. An IRR < 1 indicates a shorter LOS, whereas an IRR > 1 indicates a longer LOS. Robust standard errors (HC3) were applied as a sensitivity analysis, yielding consistent results.

Supplementary Table S4. Restricted cubic spline analysis of skeletal muscle density for prolonged LOS

Variable	Coefficient	Standard Error	Wald Z	*p*-Value
SMD (linear)	−0.0487	0.0498	−0.98	0.328
SMD (nonlinear component 1)	−0.0802	0.1103	−0.73	0.467
SMD (nonlinear component 2)	0.4812	0.6394	0.75	0.452
BMI	−0.0995	0.0396	−2.51	0.012
Albumin	−0.6809	0.3259	−2.09	0.037
CKD (yes vs. no)	1.1340	0.5509	2.06	0.040

LOS, postoperative length of stay; SMD, skeletal muscle density; BMI, body mass index; CKD, chronic kidney disease.

SMD and BMI are expressed per 1-unit increase; albumin is expressed per 1 g/dL increase; CKD is a binary variable. Restricted cubic spline analysis with four knots was used to evaluate the linearity assumption of skeletal muscle density in the logistic regression model. The overall association between SMD and prolonged LOS was significant (Wald χ^2 = 9.71, p = 0.021), whereas the nonlinear component was not significant (p = 0.752), indicating no evidence of nonlinearity.
